# Antimicrobial Drugs and Community–acquired *Clostridium difficile–*associated Disease, UK

**DOI:** 10.3201/eid1305.061124

**Published:** 2007-05

**Authors:** J. A. Chris Delaney, Sandra Dial, Alan Barkun, Samy Suissa

**Affiliations:** * McGill University Health Center, Montreal, Canada

**Keywords:** Clostridium difficile–associated disease, antimicrobial agents, fluoroquinolones, risk factors, epidemiology, dispatch

## Abstract

In a population-based case-control study of community-acquired *Clostridium difficile*–associated disease (CDAD), we matched 1,233 cases to 12,330 controls. CDAD risk increased 3-fold with use of any antimicrobial agent and 6-fold with use of fluoroquinolones. Prior use of antimicrobial agent did not affect risk for CDAD after 6 months.

Recent reports suggest that *Clostridium difficile–*associated disease (CDAD), including community-acquired CDAD, is increasing in occurrence and severity (*1*–*4*). Antimicrobial drug use is widely believed to be a key driver of CDAD infections, with differences in risk depending on class of antimicrobial agent (*2*). Differences in risk are postulated to be caused by differences in properties of the microbial agents, such as the magnitude and the duration of their effects on the fecal flora (*5*), their activity against *C. difficile,* and possibly their drug or metabolite levels in the intestinal lumen (*6*). However, most studies of CDAD and antimicrobial drugs have been hospital based. Because inpatients are often exposed to multiple antimicrobial drugs, these studies may have limited ability to evaluate agents rarely prescribed or rarely prescribed alone (e.g., macrolides).

We expand on previous work by assessing whether and to what extent the risk for community-acquired CDAD varies with the type of antimicrobial drug prescribed. We also evaluate whether and how long this effect takes to dissipate after drug discontinuation, beyond the 90-day period previously explored with this population (*3*,*4*).

## The Study

We further analyzed data from a population-based case-control study that was constructed using the United Kingdom’s General Practice Research Database (GPRD). GPRD is a well-validated (*7*) clinical database that records information taken from general practice records. The cohort used in this study has been extensively described (*3*).

Briefly, we identified all patients who had had CDAD (based on either clinical diagnosis or a positive toxin test result) from 1993 through 2004 and who were registered for ≥2 years in a general practice anywhere in the United Kingdom. Approximately 90% of tests for *C. difficile* toxin had neither a positive nor negative result in the GPRD (the result was not recorded as a variable but included as case notes that we were unable to review), and so a clinical diagnosis often indicates a test result that was not available to the investigators. Case-patients were defined as patients with community-acquired CDAD, that is, patients who had not been not hospitalized during the year before their CDAD diagnosis. Each case-patient was matched by practice and age (±2 years) to 10 control-patients who also had not been hospitalized during the prior year. Control-patients were also registered in the GPRD. By matching these control-patients to specific case-patients, we could assess the antimicrobial use in the source population from which the case-patients arose. Control-patients had the same index date as the case-patient to which they were individually matched, which enabled us to account for changing drug patterns and disease rates over time in this database. We used the British National Formulary to define the following antimicrobial drug classes: penicillins, cephalosporins and other β-lactams, tetracyclines, macrolides, sulfonamides and trimethoprim, fluoroquinolones, and all others.

A total of 1,233 case-patients were matched to 12,330 control-patients. The results of antimicrobial exposure, by drug class, are presented in Table 1. The adjusted odds ratio (OR) for CDAD with use of any antimicrobial drug in the 90 days before the index date was 3.1 (95% confidence interval [CI]: 2.7–3.6). Adjusted ORs for different classes of antimicrobial drugs were as follows: tetracyclines 0.85, sulfonamides 1.88, penicillins 1.89, macrolides 2.15, cephalosporins 2.21, and fluoroquinolones 6.20. The mean number of antimicrobial classes for those who received the drugs was 1.4 for case-patients and 1.2 for control-patients. With respect to patients who received at least 1 antimicrobial drug, 67% of case-patients and 82% of control-patients received only 1 class of antimicrobial drug (compared with 50% of case-patients and 59% of control-patients among fluoroquinolone users who received only a single class of antimicrobial drug).

**Table 1:** Antimicrobial drug exposure of patients with and without *Clostridium difficile*–associated disease, UK, 1993–2004*

Antimicrobial drug received, past 90 d	Case-patients, n = 1,233 (%)	Control-patients, n = 12,330 (%)	Crude OR†	Adjusted OR‡ (95% CI)
Any	456 (37)	1649 (13)	5.0	3.7 (3.1–4.4)
Tetracyclines	17 (1.4)	106 (0.9)	1.0	0.9 (0.5–­1.5)
Penicillins	202 (16.4)	790 (6.4)	2.4	1.9 (1.6–­ 2.4)
Sulfonamides and trimethoprim	71 (5.7)	236 (1.9)	2.3	1.9 (1.5–2.7)
Macrolides	80 (6.5)	219 (1.7)	2.7	2.2 (1.7–3.1)
Cephalosporins and other β-lactams	76 (6.2)	207 (1.7)	2.9	2.2 (1.7–3.2)
Fluoroquinolones	70 (5.7)	84 (0.7)	10.9	6.2 (4.4­– 8.8)

*OR, odds ratio; CI, confidence interval. †Adjusted for other antimicrobial drugs and prior antimicrobial drug use to ensure that all comparisons used the same reference group. ‡Adjusted for inflammatory bowel disease, diverticular disease, peptic ulcer disease and gastroesophageal reflux disease, *Helicobacter pylori*–associated disease, pernicious anemia, cancer including solid tumor and hematologic malignancies, diabetes mellitus, chronic obstructive pulmonary disease, cirrhosis, nonsteroidal anti-inflammatory agents, aspirin, H2 blockers, proton pump inhibitors, and antimicrobial drug use in the past 2 years.

Table 2 describes the residual effects after discontinuation of antimicrobial agents, fluoroquinolones in particular, as a function of the time since the last prescription. The risk (OR 3.1, CI 2.7–3.6) with current antimicrobial drug use diminished after 3 months, dropped to OR 1.8 (95% CI 1.4–2.3), and essentially disappeared after 6 months (OR 1.3, 95% CI 1.0–1.6). A similar pattern was observed after discontinuation of fluoroquinolone use. This is much lower than the effect in the 90-day window before diagnosis with CDAD.

**Table 2:** Most recent prescription for any antimicrobial drug and effect of proximity on risk of acquiring *Clostridium difficile–*associated disease, UK, 1993–2004*

*Exposure to antimicrobial drug*	*Case-patients, n = 1,233 (%)*	*Control-patients, n = 12,330*	*Crude OR*	*Adjusted OR (95% CI)†*
None (reference)	379 (30)	6,449 (52)	1.0	1.0 (reference)
Most recent prescription‡				
1–90 d (current)	456 (37)	1,649 (13)	5.0	3.7 (3.1–4.4)
91–180 d	128 (10%)	1067 (9)	2.2	1.8 (1.4–2.3)
181–365 d	131 (11)	1,498 (12)	1.6	1.3 (1.0–1.6)
1–2 y	139 (11)	1,674 (13)	1.5	1.3 (1.0–1.6)
Most recent fluoroquinolone prescription†				
1 – 90 d (current)	70 (5.7)	84 (0.7)	10.9	6.2 (4.4–8.8)
91–180 d	12 (1.0)	70 (0.6)	1.7	1.2 (0.6–2.3)
181–365 d	27 (2.2)	114 (0.9)	2.4	1.7 (1.1–2.7)
1–2 y	36 (2.9)	198 (1.6)	1.9	1.3 (0.9– 2.0)

*OR, odds ratio; CI, confidence interval. †Adjusted for inflammatory bowel disease, diverticular disease, peptic ulcer disease and gastroesophageal reflux disease, *Helicobacter pylori*–associated disease, pernicious anemia, cancer including solid tumor and hematologic malignancies, diabetes mellitus, chronic obstructive pulmonary disease, cirrhosis, nonsteroidal anti-inflammatory agents, aspirin, H2 blockers, proton pump inhibitors, and antimicrobial use in the past 2 years. ‡2 y before the index date.

To reduce the risk for protopathic bias (*8*), we did a sensitivity analysis in which we considered any patient who had a recent (90-day) diagnosis of infectious diarrhea to be unexposed to fluoroquinolones. This analysis reduced the size of the adjusted OR for fluoroquinolone use from 6.2 (95% CI 4.4–8.8) to 5.0 (95% CI 4.4–7.2).

We also conducted sensitivity analyses based on the source of the CDAD diagnosis (test-based or clinical only). These analyses showed a similar effect for exposure to any antimicrobial agents for clinical and test-based diagnoses. Among antimicrobial classes, although fluoroquinolones appear to have higher ORs in the test-based group (OR 6.7; 95% CI 4.5–10.0) than in the group with clinically based diagnoses (OR 5.2; 95% CI 2.6–0.6), this difference is not statistically different. All other antimicrobial agents were stronger risk factors for development of CDAD when the definition of CDAD was determined by clinical diagnosis alone, without a toxin-positive test result.

## Conclusions

Almost all antimicrobial drugs were associated with increased risk for community-acquired CDAD. The risk associated with fluoroquinolones was particularly elevated, as has been found in other recent studies on CDAD in hospital settings (*2*,*9*–*11*).

Because broad-spectrum antimicrobial drugs are more commonly prescribed for patients with more severe infections, the underlying indications for fluoroquinolone prescription could also be contributing to the increased risk for CDAD. That is, channeling of antimicrobial drugs toward sicker patients may result in confounding by indication (*12*). However, the sharp reduction in residual risk among fluoroquinolone users suggests that any such health status confounding is likely to be minor. Although the residual effects of antimicrobial prescriptions never drop to zero (which may indicate a minor effect of confounding), they drop significantly and quickly. This does not exclude the possibility of confounding by indication due to the infection for which the fluoroquinolone was prescribed.

In addition, our sensitivity analysis suggested some degree of robustness of this result because of misclassification, from either misdiagnosis of CDAD as another form of diarrhea or from the source of the diagnosis. However, despite the high sensitivity and specificity seen in studies that use database codes to identify CDAD cases (*13*) and the successful use of this approach in other studies (*14*,*15*), more validation work on this endpoint remains to be done to completely describe the process of recording community-based CDAD diagnoses.

Our results suggest that antimicrobial drugs are a risk factor for CDAD, including community-acquired CDAD. Despite the high risk that appears to be associated with fluoroquinolone use, only 7% of the case-patients in this sample were exposed to a fluoroquinolone, and only 37% were exposed to any class of antimicrobial drug. Therefore, while good prescribing practices for antimicrobial drugs should continue to be encouraged, these drugs are unlikely to be the primary driver of community-acquired CDAD infections in this population.

A.B. is a consultant for AstraZeneca Inc and Altana Pharma Inc. This study was funded by the Canadian Institutes of Health Research (CIHR) and the Canadian Foundation for Innovation. S.D. is a Chercheur-Boursier Clinicien and A.B. a Chercheur National awardee, both from the Fonds de la Recherche en Santé du Québec. S.S. is the recipient of a Distinguished Investigator award from CIHR**.**
